# Corioamnionitis por *Ureaplasma parvum*: a propósito de un caso

**DOI:** 10.1515/almed-2022-0103

**Published:** 2023-02-09

**Authors:** Antonio Moreno-Flores, María Domínguez-Landesa, María Guadalupe Vázquez-López, Laura Sante-Fernández

**Affiliations:** Hospital Universitario Lucus Augusti, Lugo, España

**Keywords:** corioamnionitis, parto prematuro, *Ureaplasma*

## Abstract

**Objetivos:**

Las especies de *Ureaplasma* son los microorganismos más frecuentemente relacionados con casos de parto prematuro espontáneo, rotura prematura de membranas o corioamnionitis.

**Caso clínico:**

Gestante de 28 + 6 semanas, sin antecedentes personales de interés, que acude al hospital por contracciones. Ante la sospecha clínica de corioamnionitis ingresa para cesárea segmentaria transversa, transcurriendo sin nada reseñable y siendo dada de alta a los siete días del ingreso. El neonato permanece estable y sin datos clínicos de infección. No obstante, ante la sospecha clínica de corioamnionitis se pauta tratamiento empírico con ampicilina (2 g/6 h) y gentamicina (5 mg/kg/24 h) intravenosas y se toman muestras de exudados faríngeo/amigdalar, ótico y anal/rectal. A las 24 horas se detecta en todas las muestras *Ureaplasma parvum*, suspendiéndose el tratamiento empírico e iniciando azitromicina intravenosa (12 mg/24 h). También se detecta *U. parvum* en muestra de exudado endocervical y de placenta. Tras 52 días de ingreso el lactante es dado de alta.

**Conclusiones:**

Aunque parece clara la relación entre la colonización por estos microorganismos y el desarrollo de enfermedad perinatal, el elevado porcentaje de colonización vaginal existente y el hecho de que la mayoría de embarazadas colonizadas den a luz a término, sin ningún tipo de complicación, hacen necesarios más estudios.

## Introducción

*Ureaplasma urealyticum* y *Ureaplasma parvum* son colonizadores frecuentes del tracto genital humano, siendo los microorganismos más frecuentemente aislados en líquido amniótico y placenta tras un parto prematuro [1], [2], [3], [4], [5]. No obstante, aunque parece clara la relación entre colonización e infección perinatal, un porcentaje significativo de embarazadas que están colonizadas no desarrollan ningún tipo de manifestación clínica de infección [1], [2], [3], [4].

## Caso clínico

Presentamos el caso de una gestante de 28 + 6 semanas, sin antecedentes personales de interés y con embarazo controlado, que acude al hospital por contracciones. En la analítica en sangre se observa leucocitosis (21.030/mm^3^) con un 91.1% de neutrófilos. Ante la sospecha clínica de corioamnionitis y la presentación podálica del feto se decide ingreso para cesárea segmentaria transversa, previa administración empírica de piperacilina/tazobactam (4 g/0.5 g/6 h) y claritromicina (500 mg/12 h) intravenosos, además de una única dosis de cefazolina (1 g) como profilaxis previa a la cirugía. Previo al inicio del tratamiento antibiótico, se remite al laboratorio de microbiología una muestra de exudado vaginal para su cultivo, informándose el crecimiento de una flora saprófita habitual. La intervención quirúrgica se produce sin nada que reseñar, evolucionando la paciente favorablemente y suspendiéndose el tratamiento antibiótico tras completar 48 h afebril, siendo dada de alta a los siete días del ingreso. El neonato permanece estable respiratoria y hemodinámicamente, afebril desde el punto de vista infeccioso y sin datos clínicos de infección. No obstante, ante la sospecha clínica de corioamnionitis se pauta tratamiento empírico con ampicilina (2 g/6 h) y gentamicina (5 mg/kg/24 h) intravenosas y se toman muestras de exudados faríngeo/amigdalar, ótico y anal/rectal, que son remitidas al laboratorio de microbiología para detección molecular de microorganismos de transmisión sexual (Allplex™ STI Essential Assay, Seegene Inc). A las 24 h se detecta en todas las muestras *U. parvum*, suspendiéndose el tratamiento empírico e iniciando azitromicina intravenosa (12 mg/24 h). También se detecta *U. parvum* en muestra de exudado endocervical y de placenta. Dada su buena evolución, tras 52 días de ingreso el lactante es dado de alta.

## Discusión

La corioamnionitis es la inflamación de las membranas fetales y puede estar causada por la invasión de microorganismos en la cavidad amniótica [4, 6]. Pese a ser colonizadores habituales del tracto genital, las especies de *Ureaplasma* son los microorganismos más frecuentemente relacionados con casos de parto prematuro espontáneo, rotura prematura de membranas o corioamnionitis [1, 2, 5, 7], siendo *U. parvum* la especie que invade con mayor frecuencia la cavidad amniótica [1, 3]. En el neonato pueden causar displasia broncopulmonar, enterocolitis necrosante, retinopatía prematura o lesiones cerebrales, entre otros cuadros clínicos [1, 2, 8, 9]. *Ureaplasma* spp. posee varios factores de virulencia, pero ninguno de ellos ha podido relacionarse directamente como causa de parto prematuro [3]. Varios estudios muestran al antígeno en bandas múltiples como un posible inductor de la respuesta inmune del huésped, provocando la producción de citoquinas que podrían favorecer el inicio de contracciones y, en el caso de una infección postnatal, el desarrollo de displasia broncopulmonar [3], [4], [5], [6, 8].

El hecho de carecer de pared celular hace que las especies de *Ureaplasma* sean muy sensibles a la deshidratación y a los cambios de temperatura. Además, se trata de microorganismos fastidiosos que requieren medios de cultivo complejos para satisfacer sus requerimientos nutricionales [2]. Por todo ello, las técnicas de diagnóstico molecular ofrecen ventajas en el diagnóstico, como una mayor sensibilidad y la capacidad de obtener una identificación a nivel de especie, incluso la posibilidad de cuantificar el resultado [9]. La ausencia de pared celular hace que los antibióticos betalactámicos resulten ineficaces [2, 3, 9]. Las opciones terapéuticas válidas y no teratógenas son limitadas, siendo los macrólidos la familia de antibióticos más utilizados, sobre todo azitromicina y claritromicina [2, 3]. Sin embargo, el tratamiento empírico recomendado en los casos de corioamnionitis es gentamicina combinada con ampicilina [2, 5], un tratamiento poco eficaz en estos casos [2, 5]. Por otro lado, no existe una clara evidencia científica de que las estrategias de búsqueda activa o de erradicación sean coste-efectivas, debido a que la colonización no se asocia necesariamente con el desarrollo de infección y a que los tratamientos erradicadores pueden implicar efectos adversos para la salud, aumento de resistencias antibióticas y alteraciones en el microbioma [1, 2].

En definitiva, aunque parece clara la relación entre la colonización por estos microorganismos y el desarrollo de enfermedad perinatal, el elevado porcentaje de colonización vaginal existente y el hecho de que la mayoría de embarazadas colonizadas den a luz a término sin ningún tipo de complicación hace que sean necesarios más estudios para poder comprender mejor los aspectos relacionados con el diagnóstico, tratamiento y manejo clínico de los pacientes.

## Puntos claves

– La invasión de la cavidad amniótica por microorganismos puede causar graves cuadros clínicos en el neonato.
– Las técnicas de biología molecular permiten mejorar el diagnóstico microbiológico de aquellos microorganismos que, como *U. parvum*, son difíciles de cultivar.
– Todavía son necesarios más estudios para poder comprender mejor este tipo de infecciones.
